# Impact of methane mitigation strategies on the native ruminant microbiome: A protocol for a systematic review and meta-analysis

**DOI:** 10.1371/journal.pone.0308914

**Published:** 2024-08-22

**Authors:** A. Nathan Frazier, Aeriel D. Belk, Matthew R. Beck, Jacek A. Koziel

**Affiliations:** 1 United States Department of Agriculture—Agricultural Research Service, Conservation and Production Research Laboratory, Bushland, Texas, United States of America; 2 Department of Animal Science, Auburn University, Auburn, Alabama, United States of America; Zagazig University Faculty of Agriculture, EGYPT

## Abstract

Recently, research has investigated the role of the ruminant native microbiome, and the role microbes play in methane (CH_4_) production and mitigation. However, the variation across microbiome studies makes implementing impactful strategies difficult. The first objective of this study is to identify, summarize, compile, and discuss the current literature on CH_4_ mitigation strategies and how they interact with the native ruminant microbiome. The second objective is to perform a meta-analysis on the identified16S rRNA sequencing data. A literature search using Web of Science, Scopus, AGRIS, and Google Scholar will be implemented. Eligible criteria will be defined using PICO (population, intervention, comparator, and outcomes) elements. Two independent reviewers will be utilized for both the literature search and data compilation. Risk of bias will be assessed using the Cochrane Risk Bias 2.0 tool. Publicly available 16S rRNA amplicon gene sequencing data will be downloaded from NCBI Sequence Read Archive, European Nucleotide Archive or similar database using appropriate extraction methods. Data processing will be performed using QIIME2 following a standardized protocol. Meta-analyses will be performed on both alpha and beta diversity as well as taxonomic analyses. Alpha diversity metrics will be tested using a Kruskal-Wallis test with a Benjamini-Hochberg multiple testing correction. Beta diversity will be statistically tested using PERMANOVA testing with multiple test corrections. Hedge’s *g* standardized mean difference statistic will be used to calculate fixed and random effects model estimates using a 95% confidence interval. Heterogeneity between studies will be assessed using the *I*^*2*^ statistic. Potential publication bias will be further assessed using Begg’s correlation test and Egger’s regression test. The GRADE approach will be used to assess the certainty of evidence. The following protocol will be used to guide future research and meta-analyses for investigating CH_4_ mitigation strategies and ruminant microbial ecology. The future work could be used to enhance livestock management techniques for GHG control. This protocol is registered in Open Science Framework (https://osf.io/vt56c) and available in the Systematic Reviews for Animals and Food (https://www.syreaf.org/contact).

## Background

Agriculture, forestry, and land use sectors represent 22% of the global anthropogenic greenhouse gas (GHG) emissions with enteric fermentation of methane (CH_4_) contributing 5% of the total direct contribution to global anthropogenic GHG [1]. Among global agricultural food systems, CH_4_ emissions from livestock operations accounts for 30–40% of the total global anthropogenic GHG emissions [2]. Enteric fermentation, primarily from beef and dairy cattle, represents 46% of the total livestock generated CH_4_ emissions [2]. Due to a growing world population, an increase in demand for food products that come from ruminant livestock could be seen (i.e., meat, meat products, dairy products, etc.). An increase in ruminant livestock production could therefore lead to an increase in ruminant based CH_4_ emissions, resulting in an increase in anthropogenic GHG. Additionally, due to the relatively short atmospheric lifespan of CH_4_, its mitigation has been suggested as the most promising means to limit climate warming in the short-term [3–5]. To combat the anthropogenic-livestock effects on climate change, CH_4_ mitigation strategies have been explored via dietary manipulation, rumen manipulation, and animal breeding [6].

Promising enteric CH_4_ mitigation strategies and techniques include the use of inhibitory compounds such as 3-nitrooxypropanol (3-NOP; [7]) and halogenated bromoform from seaweed [8]; red algae [9]; plant secondary compounds [10, 11], early-life microbiome engineering [12]; supplemental feed to grazing cattle [13–16]; changes to the diet composition [17]; and breeding and genetics programs designed to select for low-emissions animals [18]. Interestingly, researchers have turned their attention to understanding how these mitigation strategies affect ruminant microbial ecology. To date, several studies have investigated how various CH_4_ mitigation techniques impact the rumen microbiome including inhibitory compounds such as 3-NOP [19] and halogenated bromoform from algae [20, 21], dietary manipulation [22, 23], breeding programs [24, 25], and early-life microbiome manipulation [12].

Currently, there remains a gap in the literature for an updated comprehensive and systemic review and meta-analysis on the effects of CH_4_ mitigation strategies on the ruminant microbiome. One of the reasons for this is the variation between microbiome datasets makes comparisons between studies difficult. This variation stems from differences in sampling procedures, a lack of consensus in computational methods, and differences in preprocessing methods [26, 27]. However, compiling multiple 16S rRNA amplicon sequencing datasets and analyzing them together could elucidate key large-scale patterns and results. Therefore, the results from the proposed study are crucial to increasing our understanding of how current CH_4_ mitigation strategies influence the rumen microbiome and how the native microbiome could be used for CH_4_ reduction. Our results could also enhance on-farm guidelines for future management decisions on best practices for reducing livestock GHG emissions.

Our overall objective is to address this knowledge gap by generating a systematic review and meta-analysis identifying CH_4_ mitigation strategies that impact the rumen microbiome. To accomplish this, we present this protocol that will accomplish two preliminary aims: 1) to outline how we will identify, compile, summarize, and discuss the current literature on CH_4_ mitigation strategies and their effects on the native rumen microbiome; and 2) to detail how we will perform a meta-analysis on the selected studies from the literature curation. The future study will address the research question of how CH_4_ mitigation strategies influence the rumen microbiome of post-weaned cattle.

## Methods

**Fig 1:**
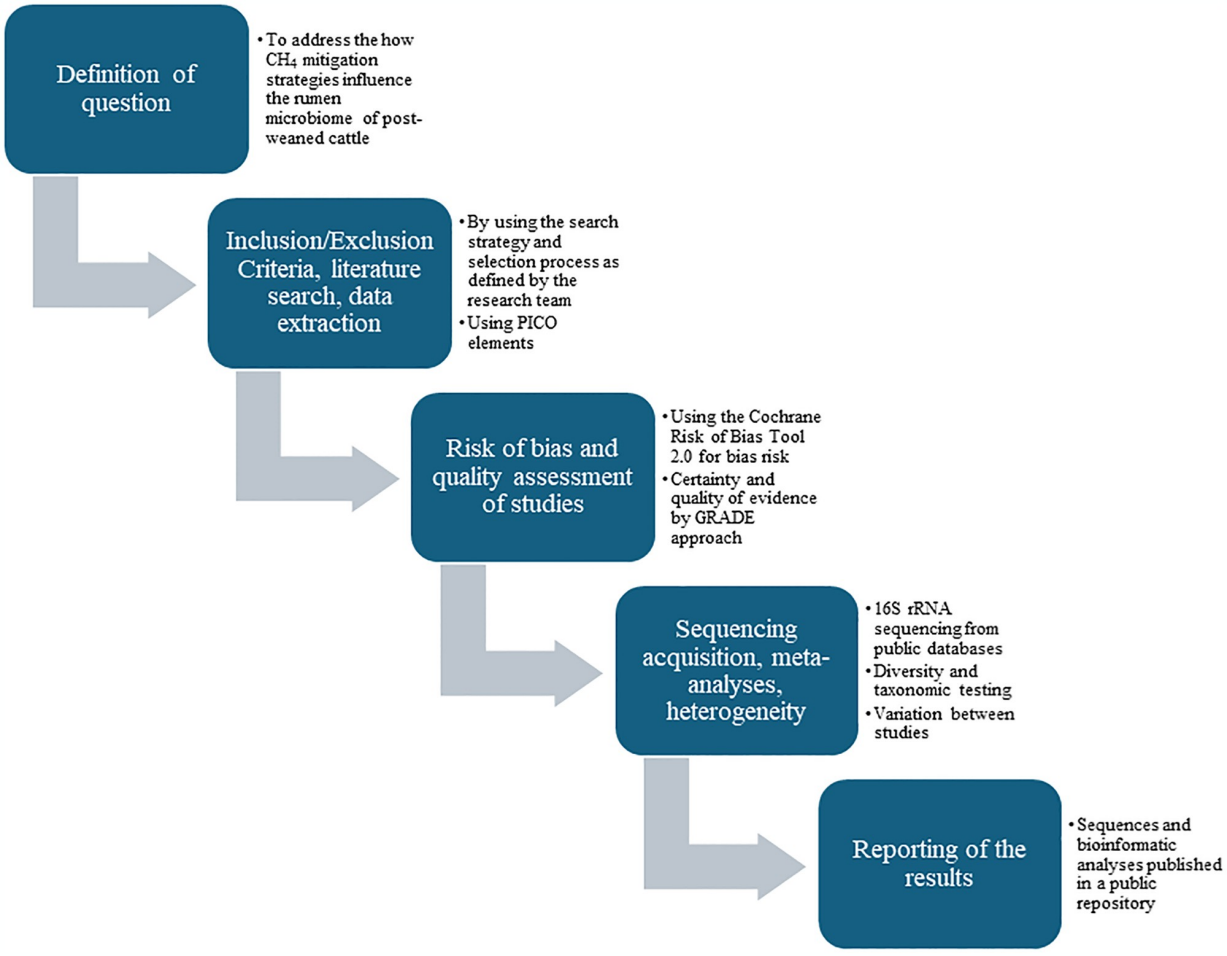
Above is a brief flowchart of the steps to be performed in the future systematic review and meta-analysis study.

Reporting of this protocol is in accordance with the Preferred Reporting Items for Systematic Review and Meta-Analysis Protocols (PRISMA-P) statement [28]. The PRISMA-P checklist is used to guide researchers while writing the protocol by providing the essential and minimum component requirements. The PRISMA-P checklist is included in S1 Table. The protocol is registered in Open Science Framework (https://osf.io/vt56c) and available in the Systematic Reviews for Animals and Food (https://www.syreaf.org/contact). The proposed systematic review and the meta-analysis will follow the recommendations of the Cochrane Collaboration Handbook for Systematic Reviews [29]. The Cochrane method is a transparent and reproducible method for scientific investigation. The future meta-analysis will correspond to the steps outlined in Fig 1.

### Eligibility criteria

The eligibility criteria were defined using Population, Intervention, Comparison, Outcome Study Design (PICO) elements (Table 1).

**Table 1:** The eligibility criteria as defined by the PICO elements.

Category	Inclusion	Exclusion
Population	Post-weaned cattle (> 6 mo.)	Pre-weaned calves
Intervention	Dietary and rumen manipulation CH_4_ mitigation strategies	Animal and feed management strategies
Comparator	Studies demonstrating significant changes in microbial ecology as well as significant CH_4_ reduction between control and treatment groups	Either no difference in microbial composition or no significant CH_4_ reduction
Outcomes	Taxonomic and diversity changes where CH_4_ reduction is seen	
Sequencing Protocol	Earth Microbiome Project Protocols will be prioritized	
Sequencing Region	16S rRNA SSU V4 or V4-V5	

### Study design, characteristics, and population

The design of the systematic review is a thorough literature search for primary research studies wherein 16S rRNA gene amplicon datasets are publicly available in a national database such as the National Center for Biotechnology (NCBI), European Bioinformatics Institute (EBI), or similar [30, 31]. Eligible studies will also be determined based on the CH_4_ mitigation strategy used with the target strategies being dietary interventions and rumen manipulations as defined by previous research [6]. Our initial search for papers will include all age ranges within post-weaned cattle (six months of age or older) to determine our population and the availability of published studies that meet our inclusion criteria. Pre-weaned cattle will be initially excluded as to not confound the microbiome datasets found within the literature review. If the number of studies found is insufficient, the parameters will be widened to include pre-weaned cattle and the appropriate measures in analysis will be taken accordingly. No restrictions will be placed on breed or sex as we are not comparing microbiomes between different breeds or sex; instead, we aim to address how CH_4_ mitigation strategies alter the rumen microbiome regardless of these parameters. Only studies published in English will be included.

Additionally, studies that meet the above criteria must also meet certain amplicon sequencing method criteria. Eligible studies must adhere to the best of their abilities to the criteria within the Earth Microbiome Project protocol [32]. These criteria include but are not limited to 16S rRNA amplicon sequencing that use primers that target the V4 or V4-V5 region of the 16S small subunit rRNA; using Illumina sequencing technology; and reports amplicons at least 390 base-pairs in length.

### Intervention, comparison groups, and outcomes

All dietary and rumen manipulation CH_4_ mitigation strategies will be included in the initial search for the intervention group. Eligible studies must have investigated the effects of CH_4_ mitigation strategies on the native rumen microbiome with no restriction for the method of inhibition, dose, or concentration. No limits will be placed on the length of the study. Our comparison group will include studies that have a significant change in microbial ecology from the control group. Additionally, studies must include outcomes where significant CH_4_ reduction was seen between control and treatment groups as well as microbial analyses. The main outcomes will include alpha and beta diversity changes as well as taxonomic profile differences between studies where CH_4_ reduction was seen.

### Search strategy

Our search strategy will include structured terms based on our PICO elements. The primary bibliographic databases to be used will include Web of Science, Scopus, International Information Systems for Agricultural Sciences and Technology (AGRIS), and Google Scholar. A preliminary literature search was conducted in October 2023 and the results of this search are described in S1 File. The preliminary search allowed us to refine our search terms which utilize the Boolean operators “AND” and “OR” to connect the search terms. The strategy for the preliminary search was “ruminant OR rumen OR ruminants OR cattle AND methane AND inhibition AND microbiome”. The search strategy to be used in the literature search stage of the study will use the above strategy with refinements used as necessary. These refinements might include but are not limited to the use of synonyms to the keywords, the use of various spellings of keywords (i.e., British versus American spelling in Standard English), and the addition of other keywords not yet listed.

### Selection and screening process

Two independent reviewers (ANF and ADB) will be responsible for carrying out the literature screening process. Each reviewer will independently screen the above-mentioned databases using the defined search strategy in two stages. The first stage will include screening the titles and abstracts of identified articles for descriptions detailed in the PICO elements. The second stage will examine the text of the studies for the PICO elements and any study not meeting the inclusion criteria will be excluded and cataloged at this stage. If at any point there is a discrepancy between the two independent reviewers during the two-staged literature screening process, a third independent reviewer will be responsible for determination of study eligibility.

### Data extraction and management

The selected eligible studies will be cataloged, and metadata will be managed using Microsoft Excel. Data extraction will be done by one reviewer and a second reviewer will be responsible for assuring data accuracy and completeness of the data collection to avoid measurement bias. The study-level data that will be extracted will include the article title, the authors’ names, the article DOI, year of publication, journal title, geographic location, the bioproject database, the bioproject number, and the study type (i.e., *in-vitro* v. *in-vivo*). Population-level data will include animal species, animal breed, animal sex, herd size, housing or pen information, feed type, methane collection system, and if applicable the *in-vitro* system used. Intervention and comparator level data will include the description of the CH_4_ intervention used, the concentration and/or dosage, and the length of the trial or study. Finally, outcome level data will include the hypervariable region(s) sequenced, primer sets, the sequencing platform, sequencing geographic location, sequencing analysis pipeline and version number, and the number of reads sequenced in the study.

### Risk of bias and quality assessment

Quality and bias risk will be assessed by one reviewer using the Cochrane Risk of Bias Tool 2.0 (RoB 2) with adaptations to the signal questioning to fit animal science studies as previously described [33, 34]. For example, questions that consider if participants are aware of their inclusion of the study will be either dropped or amended as the population of interest are ruminant livestock animals. A precedent has been described for this amendment to RoB 2 signaling questioning in previous livestock studies [34, 35]. The five domains of bias to be evaluated include the randomization process, deviations from intended interventions, missing outcome data, measurement of outcome data, and the selection of reported results. A preliminary analysis will be conducted to ensure that the criteria can be applied appropriately and consistently. Once the preliminary analysis is completed, risk assessment will be conducted by one reviewer with a second reviewer responsible for analyzing the results of the assessment for criteria application consistency. If needed, a third reviewer will be consulted to resolve any discrepancies between the first two reviewers in the process. The evaluation will be scored according to the criteria established in the RoB 2 worksheet (S2 File) and will address each domain based on “low risk”, “high risk” or “some concern for risk” of bias in each study. The overall evaluation of the article will be scored according to the following points system: between 7–10 points means there is low risk of bias; between 3–6 points indicates there is some concern for risk of bias; and below 2 points is considered high risk for bias. All articles will then be ranked according to their risk of bias evaluation score, and the level of bias will influence the degree of importance for each study in evidence synthesis.

### Data synthesis and meta-analysis

The certainty and quality of evidence will be assessed by two independent reviewers (ANF and ADB) using the Grading of Recommendations, Assessment, Development and Evaluations (GRADE) approach [36, 37]. GRADE is used to evaluate the certainty of evidence across the PICO elements. The parameters used in the GRADE method include risk of bias, imprecision, inconsistency, indirectness, and publication bias. Given any discrepancies between the two independent reviewers regarding the results of the GRADE assessment, a third reviewer will be consulted for resolution to evidence evaluation.

If there are sufficient studies (i.e., more than three) having similar definitions of the PICO elements, meta-analyses will be conducted. If in the case that pre-weaned cattle were included due to insufficient studies of post-weaned cattle, meta-analyses will be performed adjusting for age within the microbial analysis. Publicly available 16S rRNA amplicon sequencing data will be extracted using NCBI’s Sequence Read Archive using SRA Toolkit [38], QIIME2’s *fondue* plugin [39], or other extraction methods as necessary. Data processing will be performed using a modified protocol adapted from previously published methods [26, 40]. Briefly, the catalogued 16S rRNA gene datasets will be initially processed using a standardized protocol in QIIME2 version 2023.9 [41]. Demultiplexed sequences will undergo quality control using the DADA2 plugin [42] or DEMUX plugin [41] depending on original sequence quality. The resulting feature table will be filtered for mitochondria and chloroplasts. Taxonomic analyses will be assessed at the genus level by first assigning taxonomy to the amplicon sequence variants (ASVs) using the pre-trained SILVA 138 99% database via the q2-feature-classifier plugin [43, 44]. Phylogenetic diversity analyses will be conducted by creating a phylogenetic insertion tree using the q2-fragment-insertion plugin, rarefying the sequences to an acceptable level, and finally using the q2-core-diversity plugin for alpha and beta diversity analyses [41, 45, 46].

For diversity testing, Shannon’s Diversity Index [47], Faith’s Phylogenetic Diversity [48], and richness [49] will be assessed for alpha diversity and tested statistically using the Kruskal-Wallis test with a Benjamini-Hochberg multiple testing correction [50]. Meta-analyses of the above alpha diversity metrics will be performed in RStudio using the packages meta and metafor following previously published protocols [40, 51]. Hedge’s *g* standardized mean difference statistic will be used to calculate fixed (i.e., CH_4_ reduction) and random effects (i.e., difference in mitigation strategy) model estimates using a 95% confidence interval [52]. Heterogeneity between studies will be assessed using the *I*^*2*^ statistic used for calculating the percentage of variation reflecting true heterogeneity [53]. Heterogeneity varies between 0–100% which will be interpreted as follows: 0%– 39% could not be important, 40%– 59% could represent moderate heterogeneity, 60%– 90% could indicate substantial heterogeneity, and anything greater could suggest considerable heterogeneity [29, 52]. Potential publication bias will be further assessed using Begg’s correlation test [54] and Egger’s regression test [55]. Potential bias will be indicated if the number of studies is greater than 10 with a *P*-value < 0.1. For beta diversity, both weighted and unweighted UniFrac distance will be analyzed and statistically tested using PERMANOVA testing with multiple test corrections [56, 57]. All raw sequencing data used within the future study will be published to EBI, a publicly available database, using the QIITA platform [58]. The data and R scripts used for statistical analyses will be presented and described within the future manuscript as well as deposited into a GitHub repository.

## Discussion

Methane is a potent GHG with a global warming potential that is estimated to be 28-times higher than CO_2_ per unit mass on a 100-year time horizon and 82 times higher on a 20-year time horizon [59, 60]. Ruminants in agriculture, primarily dairy and beef cattle, contribute to anthropogenic GHG sources and therefore, measures have been taken to address enteric CH_4_ emissions. Recently, research has reported that manipulation of the ruminant microbiome could prove a promising avenue for CH_4_ reductions [12, 19–20, 24, 61]. Unfortunately, utilizing the information from the various microbiome studies has many technical challenges due to the variation in methodologies employed in microbial ecological studies [26, 27]. One potential work around is compiling the data from the various studies and re-analyzing the data in a more cohesive manner. Therefore, the future systematic review and meta-analysis will summarize and compile data investigating the effects of CH_4_ mitigation strategies on the rumen microbiome as well as re-analyze the compiled data from 16S rRNA amplicon sequencing studies to assess the abilities of CH_4_ mitigation strategies to manipulate the rumen microbiome. The future results from the proposed study could help both researchers and producers formulate more streamlined microbiome engineering protocols for CH_4_ reduction.

The proposed review and meta-analysis will have several strengths in that the study will follow guidelines previously reported for systematic reviews and meta-analyses studies in animal science and veterinary medicine [33, 62, 63]. Additionally, the members of the study will comprise scientists from various research backgrounds in animal science including microbiology, ruminant nutrition, food and meat safety, and agricultural engineering. The diverse nature of the research team will allow for a more robust systemic review and methodological approach. Bias will be minimized by using two independent reviewers for literature screening and data compilation. However, the study will be limited due to the various technical aspects of microbiome studies including the use of targeted primers (i.e., 16S primers), the differences in sampling methods, and the availability of sequencing data to the public. Another limitation could be only selecting studies that investigate cattle microbiomes. The data elucidated in the future study might not translate between different ruminant species. However, results could translate between ruminant species given recent evidence that there is a shared and heritable core ruminant microbiome irrespective of species [64]. Still, our preliminary search has yielded papers that meet our requirements and therefore indicate we are likely to have success with this study.

## Supporting information

S1 Table PRISMA-P (Preferred Reporting Items for Systematic review and Meta-Analysis Protocols) 2015 checklist: Recommended items to address in a systematic review protocol. (DOC)

S1 File Preliminary literature search results for data compilation performed in October 2023. (XLSX)

S2 File Revised Cochrane risk-of-bias tool for randomized trials (RoB 2): TEMPLATE FOR COMPLETION. (DOCX)
